# Supplementary figures and images for: Equine keratinocytes in the pathogenesis of insect bite hypersensitivity: Just another brick in the wall?
Source: PLoS One. 2022 Aug 1;17(8):e0266263. doi: 10.1371/journal.pone.0266263 (PMC9342730; doi:10.1371/journal.pone.0266263)

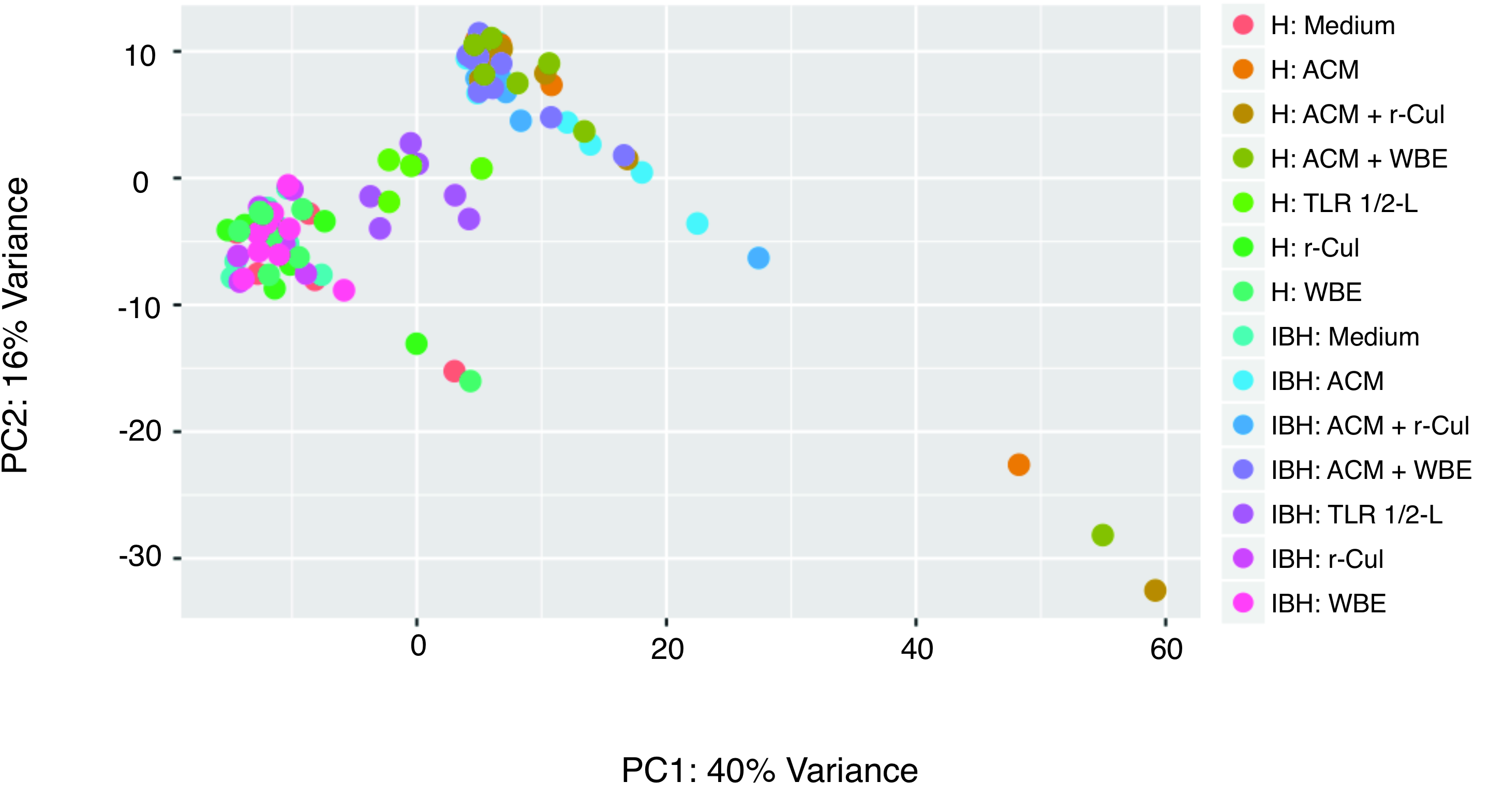

Supplement: S1 Fig — (TIF) [file pone.0266263.s001.tif]

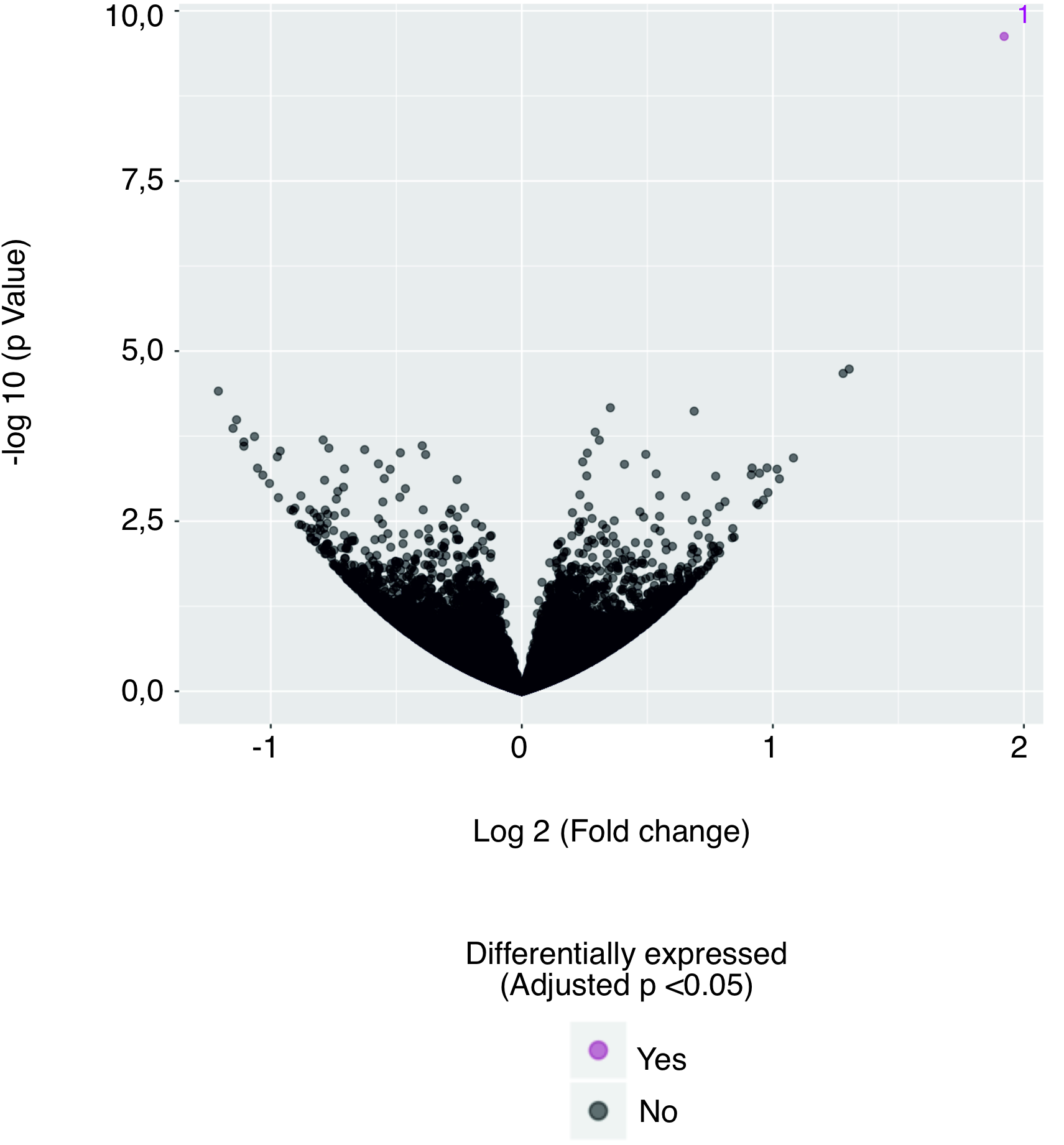

Supplement: S2 Fig — (TIF) [file pone.0266263.s002.tif]
