# Supplementary material for: Equine keratinocytes in the pathogenesis of insect bite hypersensitivity: Just another brick in the wall?
Source: PLoS One. 2022 Aug 1;17(8):e0266263. doi: 10.1371/journal.pone.0266263 (PMC9342730; doi:10.1371/journal.pone.0266263)
Supplement: Attachment — Submitted filename: Response to reviewers.docx [file pone.0266263.s018.docx]

PONE-D-21-13837
Equine keratinocytes in the pathogenesis of insect bite hypersensitivity: just another brick in the wall?
PLOS ONE

Dear Editor, dear Reviewers,

We thank the reviewers and editor for their useful comments. We have corrected the manuscript accordingly. Below please find our response to the reviewer with the corresponding line numbers in the revised manuscript marked in red.

1. Please ensure that your manuscript meets PLOS ONE's style requirements, including those for file naming. The PLOS ONE style templates can be found at https://journals.plos.org/plosone/s/file?id=wjVg/PLOSOne_formatting_sample_main_body.pdf and <https://journals.plos.org/plosone/s/file?id=ba62/PLOSOne_formatting_sample_title_authors_affiliations.pdf>.

Thank you for the comment, we think that the manuscript meets PLOS ONE's style requirements.

2. In your Methods section, please provide additional details regarding participant consent from the owners of the animals. In the ethics statement in the Methods and online submission information,

please ensure that you have specified (1) whether consent was informed and (2) what type you obtained (for instance, written or verbal). If the need for consent was waived by the ethics committee, please include this information.

The samples were all taken from slaughter horses, except for one horse, for which written consent was obtained. This information is now provided in lines 138-139.

3. We note that the grant information you provided in the ‘Funding Information’ and ‘Financial Disclosure’ sections do not match.

When you resubmit, please ensure that you provide the correct grant numbers for the awards you received for your study in the ‘Funding Information’ section.

check

4. Thank you for stating the following in the Acknowledgments Section of your manuscript:
“We thank Jelena Mirkovitch and Shui Chu Ling for their expert laboratory assistance. Arthropods used in this study were provided by the Pirbright Institute under UK under grant code BBS/E/I/00007039 awarded to Dr Simon Carpenter as part of funding received from the Biotechnology and Biological Science Research Council (UKRI).”

The Funding information has been removed from the acknowledgments.

We note that you have provided funding information that is not currently declared in your Funding Statement. However, funding information should not appear in the Acknowledgments section or other areas of your manuscript. We will only publish funding information present in the Funding Statement section of the online submission form.
Please remove any funding-related text from the manuscript and let us know how you would like to update your Funding Statement. Currently, your Funding Statement reads as follows:

Please update the Funding Statement as completed in red below:
“This work was supported by the Swiss National Science Foundation grant no. 310030-160196/1. This SNF grant was awarded to E.M. <http://www.snf.ch/>. Arthropods used in this study were provided by the Pirbright Institute under UK under grant code BBS/E/I/00007039 awarded to Dr Simon Carpenter as part of funding received from the Biotechnology and Biological Science Research Council (UKRI).

The funders had no role in study design, data collection and analysis, decision to publish, or preparation of the manuscript”

5. We note that you have stated that you will provide repository information for your data at acceptance. Should your manuscript be accepted for publication, we will hold it until you provide the relevant accession numbers or DOIs necessary to access your data. If you wish to make changes to your Data Availability statement, please describe these changes in your cover letter and we will update your Data Availability statement to reflect the information you provide.

Repository information is provided in lines 240 to 241: “The datasets generated during the current study are available in the ENA repository via accession numbers PRJEB37568 “.

6. We note that you have referenced (five r-allergens that had proven to induce sulfidoleukotriene release in IBH-affected but not in healthy horses in a basophil activation test) which has currently not yet been accepted for publication. Please remove this from your References and amend this to state in the body of your manuscript [Unpublished]”) as detailed online in our guide for authors data as supplementary material
<http://journals.plos.org/plosone/s/submission-guidelines#loc-reference-style>

We have removed this sentence from the text as we cannot provide the unpublished data. We would like to include this data in a later manuscript specific for the study that we had mentioned.

We have one new co-author, who did the new figure 1.

5. Review Comments to the Author

Please use the space provided to explain your answers to the questions above. You may also include additional comments for the author, including concerns about dual publication, research ethics, or publication ethics. (Please upload your review as an attachment if it exceeds 20,000 characters)

Reviewer #1: The papers describes the transcriptome of keratinocytes isolated from IBH- or healthy horses, non-lesional skin. The investigations and descriptions have been performed in depth, and presented in a clear manner.
The limitations of the study (no parts from lesional skin, missing recombinant allergens like proteases, no enzymatic activity testing of used WBE etc.) have been listed and discussed.

Major point:
* due to missing info for lesional skin and important allergens from Cul. saliva, and also in light of no differences between IBH and healthy horses as well as in practice not being able perform a longitudinal study (horses before and after IBH-development), I would tune down all the statements suggesting kerationcytes as being responsible in IBH development (e.g. line 25)!

**Thank you for your comment.**

**Line 43: We have written that equine keratinocytes have the ability of eliciting differing responses to different stimuli and may possibly play a role in the pathogenesis of IBH. In the next lanes we explain that they are not the primary responders but that they can amplify the allergic immune response by their activation mediated by allergic inflammatory cytokines, such as IL-4 and TFN- a.**

**Furthermore, in the discussion, lines 621-628 we address this matter and further elaborate it.**

* include in discussion the contribution of genetics/breed, environment, feed, hygiene treatment etc. of animals in IBH development

**Thank you for the comment. This is now mentioned in lines 536-544.**

* as an ACM alters transcriptome in both, IBH and healthy horses, discuss a) where that ACM might come from in a natural setting and what might be different between allergy-susceptible and healthy animals; and b) if both IBH and healthy keratinocytes react to ACM, why don't healthy develop allergies (i.e. don't they ever have a situation/setting where ACM is around, or do they have an efficient regulatory response?) ;

**Thank you for the insight. This is now added in the following lines: 92-97 and 529-536.**
Minors:

Thank you for the minor revisions. They are corrected as follows:
* short title should be short : **Corrected in line 17**
* line 37: introduce abbreviations of IHB-KER/H-KER in line 26; **line 27**
* line 38: name the single gene that differed ; **line 39**
* line 58: "...due to strong pruritus and inflicted self-trauma" ; **line 59**
* line 72: "...have been shown..."; add why E.coli expressed proteins are of limited use ;

**This is now clarified in lines 72-74: … such as endotoxin contamination and lack of post-translational modification of the protein, leading to unspecific stimulations or lack of response, respectively, and thus limiting their use for cellular *in vitro* assays ^[19]^.**

* line 76: complete with "The binding of IgE to the FceRI and crosslinking of bound IgE with allergen activates..." ;**added to line** **79**
* line 96: instead of natural model better use "a valuable source of information from horses with spontaneously ocuring disease for the role of kerationcytes in allergic skin diseases also for other species." ; **line 106-108**
* line 99: include reference, **line 110**

* line 108: Pam3CSK4: add that it is a synthetic ligand ; **line** **118**

* Methods:
- include time-point of skin sampling and medium as well as temperature for transport and storage after slaughtering; **line** **136-139**
- include stimulation conditions (conc. of stimulations, incubation time, temperature etc.); Table S1 is not sufficient; **Table S1 edited**
- line 145: which staining?; **line** **159**
- line 163: 70 or 80% confluence (stated at different places in MS); **lines 152, 158**
- throughout MS, take care to put space between "pvalues"; **corrected throughout the manuscript**
- table 4 and 5 need different titles/sub-titles

**Thank you! The titles of table 4 and 5 have been corrected**

* line 411: state in paragraph again that Cul. allergens did induce no changes, therefore were not compared between KER and H horses;

**This information is now added in lines 453-455.**

* line 422: 23 DEGs were later discussed as artefacts. **This was not well formulated and has been adapted (line 615)** - insert that info already in results section!; **has been added: lines 463-464**
* line 453: "either" "or" instead of neither/nor ; **line** **489**
* 454: "had been proven" ; **rewritten in line 491**
* line 457: again explain why E.coli expr. proteins are not suitable ; **line 490-493**
* line 459: insert "relevant" allergens ; **line 493**
* lines 461-463: change order of sentences ; **line 495-498**
* line 488: mimicking an allergic cytokine milieu with IL-4 and TNF-alpha: discuss again where that would come from in IBH horses vs. healthy ; **line** **531-533**
* line 500: "interestingly, in the present study upon stimulation..." **line** **556**
* line 515: should be sciellin***;* line 574 ***** Figures: please put in addition to figure legend the labels of the different figure parts also directly in figure for better legibility, e.g. left panels title IBH-KER vs. right panel titles IBH-H, and rec. allergens vs. WBE vs. ACM in Fig.2; like done in Fig. 4;

**This was now corrected in figures 3A-F, 4A-B, 6 A-B, 7 A-B**
* Discussion: is Filaggrin known to be important in horses as well (e.g. null-mutations)?; **no there is no evidence so far**, **line** **567-569**

Reviewer #2: Dear Authors,

The submitted manuscript describes a role for equine keratinocytes in the mechanism of allergy (specifically equine IBH) by acting as bystander responders to allergy-associated cytokines. This provides a link between immune cells and epidermal cells in amplifying an allergic response. Please see below my review comments on your manuscript.

MAJOR COMMENTS
1. Please add more background on the selection of IL-4 and TNF-a, but not others, and what is known about these two cytokines in IBH pathogenesis. Why were other Th2 cytokines, such as IL-5 and IL-13, not included? How did you determine the concentrations of the ACM?

How did you determine the concentrations of the r-allergen, WBE and TLR ligand stimulation conditions?;

**Thank you for your comment. This is added in lines 92-97 and 194-196. At the time of the experimental part of the study, equine recombinant IL-5 and IL-13 were not, unlike nowadays, available. Additionally, we wanted to focus more on IL-4, being shown to be upregulated in the skin of IBH-affected horses. However, considering the novel data from human medicine showing that IL-13 seems to be the key player in the peripheral sights (e.g. skin) of atopic individuals, and our data from sequencing full lesional skin of IBH-horses that showed upregulation of IL13, it would have been very interesting to have a group of keratinocytes stimulated with IL-13 alone or in combination with IL-4 and TNF-alpha.**

2. Please clarify the stimulation conditions in the methods. Line 165 should say “…WBE OR toll like…”.

**Thank you has been modified. Line 189**

Figure 1 suggests that some samples were co-stimulated with r-allergens or WBE and also with ACM. However, those data are not presented. Either show the data from co-stimulations or clarify Figure 1. ;

**Thank you for the valuable insight. Four supplementary tables (Table S6, -S7, -S8 and -S9) were added, and text in lines 376-379 was accordingly added.**

3. Does pre-exposing KER to r-allergens/WBE change the transcriptional response after subsequent ACM stimulation? In other words, is there an additive effect of allergen and cytokine on the KER response?

**Thank you for the comment. This is now added in lines 376-379.**

4. Add a figure validating the purity and identity of keratinocyte cultures.

**Thank you for comment. A Figure was added as Figure 1.**

5. Figures 4 and 7 are challenging to interpret with many different color codes. I suggest adding FDR/p(adj) values as well. And/or change the order of genes in descending Log2 fold change so each color-coded category is grouped together. Graphical representation of these data would also be useful.
**Thank you for the comment. Figures were changed so that change of the order of genes is in descending Log2 fold change and therefore each color-coded category is grouped together (now Figure 5 A-B and Figure 8).**

6. Please comment in the discussion how keratinocytes in lesional skin of IBH horses may differ from the keratinocyte samples compared in this study.

**Thank you for your insight. This is added in lines 514-518.**

MINOR COMMENTS

**Thank you very much for the minor comments, as well!**

1. Should line 187 say 17-31 MIL 2x50bp? Correct typo if it is one. ; **line** **213**

2. The figure titles and captions need to be improved. The first sentence should summarize the point of the figure (ex: in Fig 2 this could just be “Volcano plots of significant DEGs in different comparisons”), and the following unbolded sentences should describe the different subparts of the figure, describing sample types, color coding, etc. Currently, the figure titles are what should be the caption.

**This was changed throughout the manuscript.**

3. In Figures 2+5, it would be beneficial to label the genes of interest that are highlighted in Figures 4+7

**Unfortunately these figures are produced by a software where the labeling of genes is not possible.**
4. Line 452-453 is a double negative. Should be “did not change…either of the r-allergens….”; **line 489**

5. Line 460: activate; **rewritten, now** **line 498**

6. Line 556: artifacts ; **line** **615**

7. Line 556: outlier ; **line 616**

6. PLOS authors have the option to publish the peer review history of their article ([what does this mean?](https://journals.plos.org/plosone/s/editorial-and-peer-review-process#loc-peer-review-history)). If published, this will include your full peer review and any attached files.


If you choose “no”, your identity will remain anonymous but your review may still be made public.


**Do you want your identity to be public for this peer review?** For information about this choice, including consent withdrawal, please see our [Privacy Policy](https://www.plos.org/privacy-policy).

Reviewer #1: No

Reviewer #2: No

[NOTE: If reviewer comments were submitted as an attachment file, they will be attached to this email and accessible via the submission site. Please log into your account, locate the manuscript record, and check for the action link "View Attachments". If this link does not appear, there are no attachment files.]

While revising your submission, please upload your figure files to the Preflight Analysis and Conversion Engine (PACE) digital diagnostic tool, <https://pacev2.apexcovantage.com/>. PACE helps ensure that figures meet PLOS requirements. To use PACE, you must first register as a user. Registration is free. Then, login and navigate to the UPLOAD tab, where you will find detailed instructions on how to use the tool. If you encounter any issues or have any questions when using PACE, please email PLOS at [figures@plos.org](mailto:figures@plos.org). Please note that Supporting Information files do not need this step.
