# Supplementary material for: Equine keratinocytes in the pathogenesis of insect bite hypersensitivity: Just another brick in the wall?
Source: PLoS One. 2022 Aug 1;17(8):e0266263. doi: 10.1371/journal.pone.0266263 (PMC9342730; doi:10.1371/journal.pone.0266263)
Supplement: Attachment — Submitted filename: Response to reviewers-2nd revision.docx [file pone.0266263.s019.docx]

PONE-D-21-13837R1

Equine keratinocytes in the pathogenesis of insect bite hypersensitivity: just another brick in the wall?

PLOS ONE

Dear Editor, dear Reviewers,

Thank you for you useful comments.

We have corrected the manuscript according to the reviewer's comments. Changes are marked in light blue in the revised manuscript with track changes.

There is just one comment (major revision no 3) that we cannot address. We think that validation of the RNAseq results with qPCR analysis of lesional and/or nonlesional skin is beyond the scope of this manuscript, as we have studied the response of primary keratinocyte cultures derived from skin of IBH-affected and healthy horses following in vitro stimulations. Furthermore, a recent study showed by qPCR that IL-31 was detectable exclusively in the lesional skin of IBH-affected horses, but not detectable in the skin of healthy horses (Olomski et al., 2020). This study is cited in our manuscript (Ref no 37). Additionally, we are not able to perform further experiment because the first author of our paper is no longer working in our group and our technician is unfortunately on a longer-term sick leave. Therefore, at the moment, it would be impossible for us to perform qPCRs or other experiments. We thus hope that our manuscript will be acceptable for publication without these additional experiments.

We have made no changes to the financial and we have made no changes to the references in this revision of the manuscript.

Reviewers' comments:

Reviewer's Responses to Questions

**Comments to the Author**

1. If the authors have adequately addressed your comments raised in a previous round of review and you feel that this manuscript is now acceptable for publication, you may indicate that here to bypass the “Comments to the Author” section, enter your conflict of interest statement in the “Confidential to Editor” section, and submit your "Accept" recommendation.

Reviewer #1: All comments have been addressed

Reviewer #2: (No Response)

2. Is the manuscript technically sound, and do the data support the conclusions?

The manuscript must describe a technically sound piece of scientific research with data that supports the conclusions. Experiments must have been conducted rigorously, with appropriate controls, replication, and sample sizes. The conclusions must be drawn appropriately based on the data presented.

Reviewer #1: Yes

Reviewer #2: Yes

3. Has the statistical analysis been performed appropriately and rigorously?

Reviewer #1: Yes

Reviewer #2: Yes

4. Have the authors made all data underlying the findings in their manuscript fully available?

The [PLOS Data policy](http://www.plosone.org/static/policies.action#sharing) requires authors to make all data underlying the findings described in their manuscript fully available without restriction, with rare exception (please refer to the Data Availability Statement in the manuscript PDF file). The data should be provided as part of the manuscript or its supporting information, or deposited to a public repository. For example, in addition to summary statistics, the data points behind means, medians and variance measures should be available. If there are restrictions on publicly sharing data—e.g. participant privacy or use of data from a third party—those must be specified.

Reviewer #1: Yes

Reviewer #2: Yes

5. Is the manuscript presented in an intelligible fashion and written in standard English?

PLOS ONE does not copyedit accepted manuscripts, so the language in submitted articles must be clear, correct, and unambiguous. Any typographical or grammatical errors should be corrected at revision, so please note any specific errors here.

Reviewer #1: Yes

Reviewer #2: Yes

6. Review Comments to the Author

Please use the space provided to explain your answers to the questions above. You may also include additional comments for the author, including concerns about dual publication, research ethics, or publication ethics. (Please upload your review as an attachment if it exceeds 20,000 characters)

Reviewer #1: All comments have been adequately addressed, points that could not be answered or investigated have been sufficiently discussed as limitations of the study.

Reviewer #2: The authors have addressed many of the prior concerns and revisions to their manuscript describing the role of equine keratinocytes in insect bite hypersensitivity. Thank you. They also describe the importance of cytokine signaling in developing and exacerbating the allergic response. Most of my revisions are minor grammatical corrections. I do have some major revisions regarding that should be addressed before acceptance. Please see my recommendations below.

Major revisions
1) Lines 42-44 and 620-621. These two sentences need to be changed to better describe the identity of keratinocytes. While the data support that keratinocytes are involved in the first line of immune defense, it is incorrect to state that they are “bona fide innate immune cells”. Expression of immune related genes shows that they are involved in the immune response, but calling them “innate immune cells” would mean that they are hematopoietically derived, which is not shown with the data and cannot be based on gene expression alone. Further characterization of cell ontogeny would be needed to call them this. Also, epidermal cells are involved in the immune response even though they are not immune cells.
Please modify these sentences in the abstract and discussion/conclusion to better describe the identity and role of keratinocytes in the immune response. Please remove “bona fide innate immune cells”.
Here is an example for the abstract: “Our data suggests that equine keratinocytes contribute to the innate immune response and are able to elicit responses to different stimuli, possibly playing a role in the pathogenesis of IBH.”

*Thank you very much for your comprehensive insight. The changes were adapted in the lines 45-47 and 617-618.*

2) Figures 5 and 8 are both tables and I recommend that they be turned into tables instead of figures. Below are a few specific changes that should be made:

*Thank you for your suggestion. The figures are now turned into tables.*

-Line 332: Please fix the title to read more clearly. I recommend this, or similar:
“DEGs are classified by gene families that influence (A) the immune response and (B) epithelial barrier formation and maintenance.”

*Thank you very much. Title is now fixed in 332-333.*

-Line 334-340 and 438-441: Please add to the legend more details about the experiment that was done. In the first sentence, please add that the cell samples were analyzed by RNA-seq and that gene expression was compared between IBH-KER and H-KER. This will be redundant with the figure/table text but will greatly enhance clarity and accurate interpretation.

*Thank you. The suggested changes are now implemented in lines 333-336 and 444--448.*

The line 332 and 334-3340 and
-Line 334-340: I recommend re-stating in the legend (not just the title) that (A) shows upregulated immune response genes and (B) shows upregulated epithelial barrier genes. Alternatively, you could combine the table into one table instead of two.

*Thank you! The changes are implemented in the lines 336-337.*

3) It would be helpful to validate the RNAseq results with qPCR analysis of lesional and/or nonlesional skin. This could be shown in new Figures 5+8 if the current figures become Tables. Please consider adding a graph that shows the gene expression of key genes (IL31, KRT80, etc) in the different conditions.

*In this manuscript we have investigated the response of primary keratinocytes derived from skin of IBH-affected and healthy horses following in vitro stimulations. We find that validating our findings in lesional and nonlesional skin is thus beyond the scope of the study presented here. Furthermore, a recent study showed by qPCR that IL-31 was detectable exclusively in the lesional skin of IBH-affected horses, but not detectable in the skin of healthy horses (Olomski et al., 2020). This study is cited in our manuscript (Ref no 37).*

*Additionally, we are not able to perform further experiment because the first author of our paper is no longer working in our group and our technician is unfortunately on a longer-term sick leave. Therefore, at the moment, it would be impossible for us to perform qPCRs or other experiments.*

Minor revisions
*Thank you very much. All the minor revisions are implemented in the following lines:*

Line 92: Add period/punctuation after milieu. *Line 92.*

Line 93: Introduce ACM abbreviation here and use throughout. *Line 93 and all throughout manuscript.*

Line 101: Fix grammar to say “consisting of A combination of recombinant...”. *Line 101.*

Line 102, 104: Capitalize “Type I...”. Please make sure this is correctly capitalized throughout manuscript. *Lines 102, 104 and all throughout manuscript.*

Tables 2+3: Some of the term names are cut off. Please adjust so that they fit in the table, or add full term name as a table footnote. *Implemented in both tables.*

Lines 320 + 355: Section titles are both listed as “A)”. Please fix to say “a” and “b”, or remove lettering list.
*Implemented in lines 320 (a) and 361 (b).*

Lines 324-325: Chemokine genes do not have a dash in them, according to nomenclature rules. Please fix here and also make sure they are correctly named throughout. For example, chemokine gene names should read as “CCL20”. *Lines 324-325 and throughout manuscript.*

Line 327: Please add “, the” after IBH-KER so that the sentence here is more clear:
“Interestingly, in IBH-KER, the atopic cytokine...” *Line 327.*

Line 409: Fix grammar to say “belonging to the DNA replication process...” *Line 415.*
